# Analysis of the first tear film break-up point in Sjögren’s syndrome and non-Sjögren’s syndrome dry eye patients

**DOI:** 10.1186/s12886-021-02233-6

**Published:** 2022-01-03

**Authors:** Songjiao Zhao, Qihua Le

**Affiliations:** 1 Department of Ophthalmology, Eye, Ear, Nose & Throat Hospital of Fudan University, No. 83 Fen Yang Rd, Shanghai, 200031 China; 2 Research Center, Eye, Ear, Nose & Throat Hospital of Fudan University, Shanghai, China; 3 Myopia Key Laboratory of Ministry of Health, Eye, Ear, Nose & Throat Hospital of Fudan University, Shanghai, China

**Keywords:** The first tear film break-up point, Sjögren’s syndrome dry eye, Non-Sjögren’s syndrome dry eye

## Abstract

**Background:**

Tear film instability plays an important role in the course of Sjögren’s Syndrome dry eye (SSDE) even though it is generally classified as aqueous-deficient dry eye. The measurement of the first tear film break-up point (FTBUP) helps to evaluate the most unstable position of the tear film on ocular surface. We aim to investigate FTBUP in Sjögren’s Syndrome dry eye (SSDE) and non-Sjögren’s Syndrome dry eye (NSSDE) patients, and explore its correlation with dry eye indices.

**Methods:**

Twenty-two SSDE patients (44 eyes) and 22 NSSDE patients (44 eyes) were enrolled in the study. Oculus Keratograph K5M was used to measure FTBUP, the first and average non-invasive keratographic breakup time (f-NIKBUT and av-NIKBUT), the tear meniscus height, and meibomian gland dropout. Other tests of tear film were also performed including Ocular Surface Dryness Index (OSDI), Schirmer I test, fluorescein break-up time and corneal fluorescein staining. Dry eye indices and the locations of the FTBUP were compared between SSDE and NSSDE patients. Generalized estimating equation (GEE) was used to ajusted the correlations between right and left eyes. The correlations between the FTBUP and ocular symptoms and signs were investigated using Pearson’s correlation coefficient test.

**Results:**

The FTBUP occurred at the supranasal quadrant in 12/88 eyes, supratemporal quadrant in 8/88 eyes, inferonasal quadrant in 34/88 eyes, and inferotemporal quadrant in 34/88 eyes. The percentage eyes with inferior FTBUP was significantly higher in the SSDE than in the NSSDE subjects (86.3% vs 68.1%, *P = .049*). Moreover, in SSDE subjects, temporal breakup point was seen more often in those who presented corneal fluorescein staining in any location, while nasal breakup point was more frequent in those who did not present any corneal fluorescein staining (*P = .045*).

**Conclusion:**

The location of the FTBUP in SSDE patients had specific characteristics. However, the diagnostic potential of FTBUP in early recognition of SSDE needs further validation.

## Background

Dry eye is a multifactorial disease of the ocular surface in which tear film instability and hyperosmolarity, ocular surface inflammation and damage, and neurosensory abnormalities play etiological roles [1]. Dry eye has become the focus of ophthalmologists because of its increasing morbidity and trend in young people. The tear film serves as the most anterior barrier of the ocular surface, reducing the exposure of the corneal epithelium to air and providing a smooth optical surface [2–4]. To maintain the quantity and quality of a healthy tear film, a sufficient volume of tears and normal properties of the lipid layer are necessary [5]. The loss of homeostasis of the tear film is the major characteristic of dry eye, leading to varying degrees of ocular symptoms such as dryness, grittiness and a burning sensation.

Sjögren’s syndrome is a severe autoimmune disorder that destroys exocrine glands, including lacrimal and salivary glands, with lymphocytic infiltration [6]. Sjögren’s syndrome dry eye (SSDE) is generally classified as aqueous-deficient dry eye because of the hyposecretion of lacrimal glands that were attacked by activated T-cells [7]. Other pathologic changes, such as meibomian gland dropout and mucin deficiency, are often seen in SSDE [8–10]. The clinical manifestations in SSDE are more severe than those in non-Sjögren’s syndrome dry eye (NSSDE), including worse tear secretion, shorter tear film break-up time, more intensive corneal epithelial staining, more severe ocular surface inflammation, fewer corneal nerve fibres and lower cellular density of epithelial cells [9–14]. In SS, severe complications may be developed including corneal perforation/scleritis, and other organs may also be affected. Early recognition is important in SS to prevent vision or life-threatening complications, unlike other autoimmune diseases, are first seen by eye care providers [15–17].

With the development of imaging technologies, new quantitative examinations with good repeatability have been introduced for the clinical evaluation of dry eye. The Oculus Keratograph 5 M (Wetzlar, Germany) has been suggested to be a noninvasive, valid and stable way to evaluate tear film stability and dynamics through the measurement of the first tear film break-up point (FTBUP) and noninvasive keratograph tear film break-up time (NIKBUT) [18]. Nevertheless, only a few studies have focused on the location of FTBUP in dry eyes [19, 20]. It has been reported that the peripheral domain of the inferior quadrant and the central domain of the superior quadrant were the most common FTBUP areas in patients with cataract and dry eye [21]. However, no study has compared the location of FTBUP between SSDE and NSSDE patients, and the clinical relevance of the location of FTBUP has yet to be explored. We expected that the location of FTBUP would differ between SSDE and NSSDE in terms of the differences of tear film stability and corneal epithelial lesion, which would be beneficial for the early recognition of SS. This cross-sectional comparative study evaluated FTBUP of SSDE and NSSDE using the Oculus Keratograph and investigated its correlation with other dry eye examinations.

## Methods

### Patients

Forty-four patients were recruited consecutively from the dry eye clinic at the Eye, Ear, Nose & Throat Hospital of Fudan University from March to October 2019. Written informed consent was obtained from all the participants.

The diagnosis of primary and secondary Sjögren’s syndrome was made based on the 2012 American College of Rheumatology (ACR) classification criteria [22]. The inclusion criteria for dry eye included the presence of at least one of the following dry eye symptoms (dryness, burning sensation, grittiness, photophobia, pain, tickle) and one of the following signs: ① Schirmer I test≤5 mm/5 min; ② BUT<5 s; ③ the presence of fluorescein staining with either 5 mm/5 min<Schirmer I test≤10 mm/5 min or 5 s ≤ BUT<10s. All enrolled subjects met the criterion for dry eye, and SSDE patients additionally met ACR criterion. The exclusion criteria were a history of ocular surgery within 6 months; concomitant ocular lesions such as acute inflammation or infection, glaucoma, etc.; a history of wearing contact lens; ocular trauma; eyelid abnormalities; pregnancy or lactation; systematic diseases that would probably affect tear function except primary and secondary Sjögren’s syndrome; medications that would alter the ocular surface; and patients who could not cooperate during the examinations.

Forty-four eyes of 22 SSDE patients (20 females, 2 males, average age 58.32 ± 12.11 years) and 44 eyes of 22 NSSDE patients (19 females, 3 males, average age 53.41 ± 11.78 years) met the inclusion criteria. Among the 22 SSDE patients, 20 patients were not newly diagnosed (4 months to 10 years since initial diagnosis), and 19 were receiving topical cyclosporine A eye drop treatment. None of the NSSDE patients had topical immunosuppressive medications.

### Ocular examinations

#### Order of ocular examinations

With the help of a trained interviewer, the participants completed the Ocular Surface Disease Index (OSDI) before the ocular examinations to ensure that the clinical examination would not influence their responses. After that, the Oculus Keratograph 5 M was performed. Then all subjects had slit-lamp examination, fluorescein tear film break-up time (FBUT) assessment, and the evaluation of vital staining of the ocular surface. The Schirmer I test was performed at the end. The interval between the tests was more than 15 min. The time of examination was from 9 a.m. to 12 a.m. The room was quiet with a constant temperature of 25 °C and humidity of 50%. None of the patients used any eyedrops on the day of examination.

#### Ocular surface disease index (OSDI)

The OSDI questionnaire included twelve questions to quantify ocular disability during a one-week recall period. The participants were assessed on three main subscales: ocular symptoms, vision-related functions and limitations, and environmental stimulant [23]. A higher OSDI value (0–100) indicated more severe ocular discomfort.

#### Oculus Keratograph 5 M

**Fig. 1:**
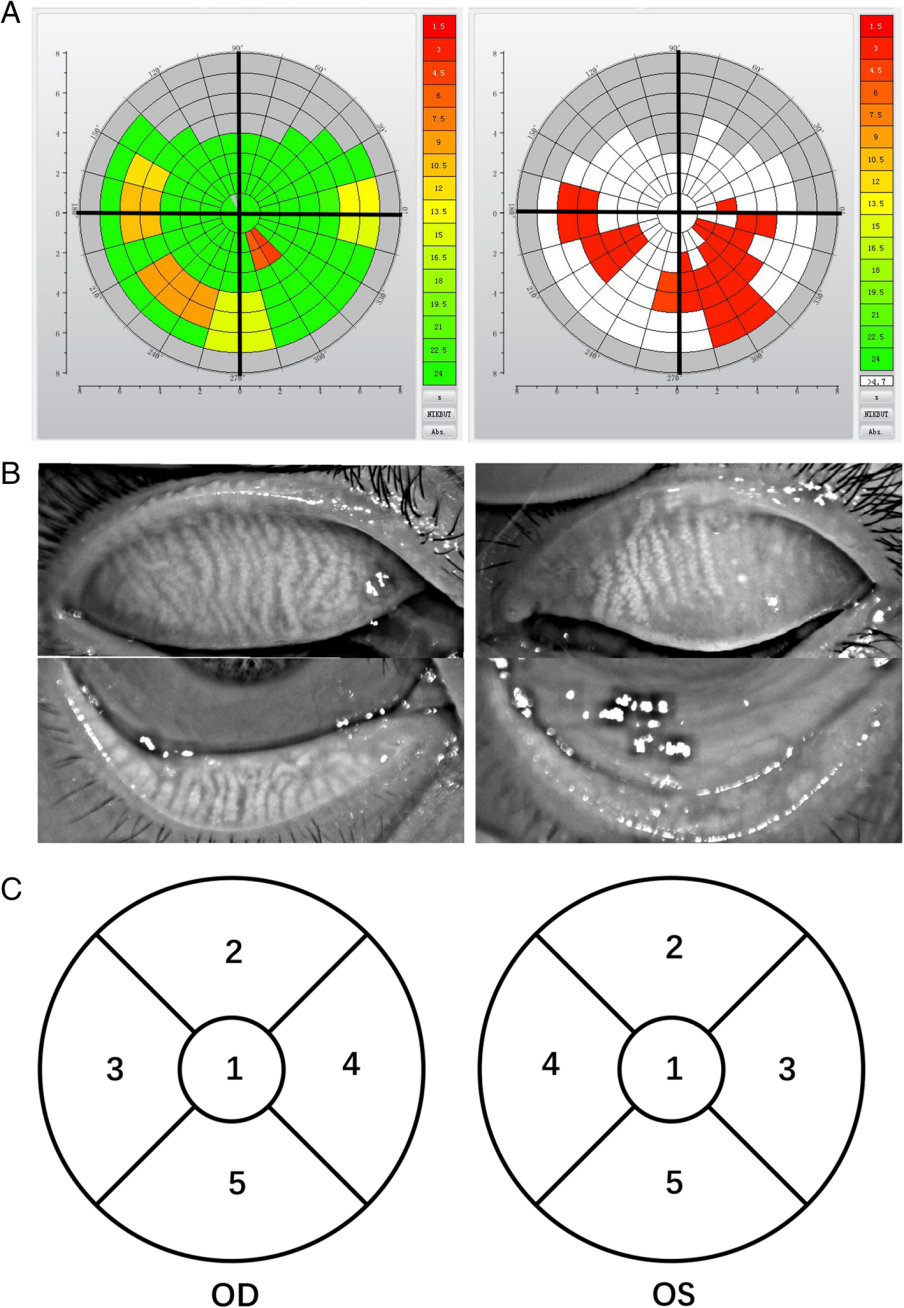
**(A)** Representative output images of the noninvasive tear film break-up points and tear break-up time measurement from a non-Sjögren’s Syndrome Dry Eye (a) and a Sjögren’s Syndrome Dry Eye (b). The colored tear map showed the position of tear break-up point and the break-up time. Red: 0–6 s; orange/yellow: 6–12 s; green: 12–24 s. (B) Representative output images of meibography from a non-Sjögren’s Syndrome Dry Eye (a) and a Sjögren’s Syndrome Dry Eye (b). The meibomian gland dropout was more severe in Sjögren’s Syndrome Dry Eye (b) than non-Sjögren’s Syndrome Dry Eye (a). (C) The cornea surface was divided into five areas when observing cornea fluorescein staining

The Oculus Keratograph 5 M equipped with modified TF-scan software was used to assess the location of FTBUP, NIKBUT, meibomian gland dropout and tear meniscus height. In a dark room condition, all subjects were asked for two blinks and then keeping eyes open as long as possible until the next blink occurred. The output image indicated the FTBUP and NIKBUT (Fig. 1A). The procedure was repeated three times for each eye. Next, all subjects were turned back upper and lower eyelids by operator to capture the meibography (Fig. 1B).

#### Slit lamp examination

All participants had a thorough examination under slit lamp biomicroscopy to exclude any other ocular abnormalities that might cause interference to tear film. Fluorescein strips (Jingming, Tianjing, China) were used to instil a minimal volume fluorescein into the lower fornix for the evaluation of FBUT and corneal vital staining during slit lamp examination.

#### Schirmer I test

The Schirmer test without anaesthesia (Schirmer I test) is a reliable and objective test to evaluate basic tear flow [1]. A Schirmer paper strip (5 × 40 mm, Jingming, Tianjing, China) was folded at the notch, and the folded end was placed into the temporal one-third portion of the lower conjunctival fornix. Patients were asked to close their eyes gently for 5 min. The length of wetting from the notch was measured.

### Image analysis and measurement

#### Assessment of FTBUP and NIKBUT

During two blinks, the regularity of the tear film surface changed when the tear film broke up. Irregularities in the image indicated places on the cornea where the tear film was losing stability or breaking up, which was captured and presented as a colour-coded tear map (Fig. 1A). Different colours indicated different tear film break-up times in different areas of cornea surface. The location with the colour closest to red was considered as the FTBUP. Meanwhile, the time between the first blink and the tear film break-up in this area was recorded as the first-NIKBUT. The mean value of the first NIKBUT obtained in three examinations were calculated as average NIKBUT.

#### Measurement of Meibography and the tear meniscus height

ImageJ software (National Institutes of Health, USA, https://imagej.nih.gov/ij/) was used to qualitatively analyse the dropout rate of meibomian glands, as previously reported [24]. The tear meniscus height was measured three times, as previously reported [25], by the use of infrared images taken at the central point of the lower lid margin. The average values were calculated.

#### Measurement of FBUT

The patients were required to blink naturally several times after the instillation of fluorescein, and then keep the eyes open and look straight ahead. Under the cobalt blue light, the time (seconds) from the last blink to the first appearance of dry spots on the corneal surface was recorded as FBUT. The examination was performed three times for each eye to time the FBUT.

#### Analysis of corneal fluorescein staining

Corneal fluorescein staining was evaluated within 1–3 min after fluorescein instillation [26]. The corneal surface was divided into five areas as proposed by the US National Eye Institute (Fig. 1C) [27], and the punctate staining in each area was recorded as a score of 0–3: 0: no staining; 1: < 15 dots; 2: 16–20 dots; 3: > 30 dots, strip/bulk staining or corneal filaments. The range of the total score was 0–15. Scores ≥1 were considered positive fluorescein staining.

### Statistics analysis

The statistical analysis was performed using SPSS software (version 24, SPSS Inc., Chicago, IL). Tabulated data are presented as the mean ± SD. Generalized estimating equation (GEE) [28] was used to adjust the correlation between right and left eyes and to compare the mean age, OSDI value, Schirmer I test, tear meniscus height, FBUT, NIKBUT, and fluorescein staining score between SSDE and NSSDE groups. The comparisons of FTBUP between SSDE and NSSDE groups and dry eye patients with or without corneal fluorescein staining was made using GEE. The differences in meibomian gland dropout between SSDE and NSSDE groups were also analysed by GEE. Pearson’s correlation coefficient test was used to analyse the relationship among dry eye-related indices in SSDE and NSSDE. A *P* value less than 0.05 was considered statistically significant.

## Results

### The location of FTBUP

In general, FTBUP occurred more frequently at the inferior cornea than at the superior cornea in both SSDE (inferior 86.3% vs superior 13.6%, *P < .001*) and NSSDE (inferior 68.2% vs superior 31.8%, *P = .014*). Moreover, the percentage eyes with inferior FTBUP was significantly higher in SSDE than in NSSDE group (*P = .049, OR = 0.338*). Further analysis showed that FTBUP of SSDE was found in the supranasal quadrant in 4 (9.1%) eyes, in the supratemporal quadrant in 2 (4.5%) eyes, in the inferonasal quadrant in 17 (38.6%) eyes, and in the inferotemporal quadrant in 21 (47.7%) eyes; in NSSDE, the number of eyes with FTBUP in each of the respective quadrants was 8(18.2%) eyes, 6 (13.6%) eyes, 17 (38.6%) eyes and 13 (29.5%) eyes. Although the distributions of FTBUP in the four quadrants in SSDE and NSSDE were not identical, no significant difference was found between the two groups (*P = .338*) (Table 1).

**Table 1:** Comparison of FTBUP between SSDE and NSSDE, and between eyes with and without fluorescein staining (n = eyes)

		Supranasal (n/%)	Supratemporal (n/%)	Inferonasal (n/%)	Inferotemporal (n/%)	Total (n/%)	*P* value	
SSDE	FL (−)	4 (26.7%)	0 (0.0%)	5 (33.3%))	6 (40.0%)	15 (100.0%)	**.045**^**a**^	.338^*∆^ .049^*‡^
	FL (+)	0 (0.0%)	2 (6.9%)	12 (41.4%))	15 (51.7%)	29 (100.0%)		
	Total	4 (9.1%)	2 (4.5%)	17 (38.6%))	21 (47.7%)	44 (100.0%)	**<.001**^**c**^	
NSSDE	FL (−)	7 (18.4%)	6 (15.8%)	14 (36.8%)	11 (28.9%)	38 (100.0%)	.215^**a**^	
	FL (+)	1 (16.7%)	0 (0.0%)	3 (50.0%)	2 (33.3%)	6 (100.0%)		
	Total	8 (18.2%)	6 (13.6%)	17 (38.6%)	13 (29.5%)	44 (100.0%)	**.014**^**c**^	
	Total	20 (22.7%)		68 (77.3%)			**<.001**^**e**^	
FL (−)	SSDE	4 (26.7%)	0 (0.0%)	5 (33.3%))	6 (40.0%)	15 (100.0%)	.865^**b**^	.039^**∆^ .011^**‡^
	NSSDE	7 (18.4%)	6 (15.8%)	14 (36.8%)	11 (28.9%)	38 (100.0%)		
	Total	11 (20.8%)	6 (11.3%)	19 (35.8%)	17 (32.1%)	53 (100.0%)	**.012**^**d**^	
FL (+)	SSDE	0 (0.0%)	2 (6.9%)	12 (41.4%))	15 (51.7%)	29 (100.0%)	.688^**b**^	
	NSSDE	1 (16.7%)	0 (0.0%)	3 (50.0%)	2 (33.3%)	6 (100.0%)		
	Total	1 (2.9%)	2 (5.7%)	15 (42.9%)	17 (48.6%)	35 (100.0%)	**<.001**^**d**^	
	Total	20 (22.7%)		68 (77.3%)			**<.001**^**e**^	

*SSDE* Sjögren’s Syndrome Dry Eye, *NSSDE* Non- Sjögren’s Syndrome Dry Eye

FL(+), the presence of fluorescein staining; FL(−), the absence of fluorescein staining

*∆: Generalized estimating equation between SSDE and NSSDE groups in four different quadrants

*‡: Generalized estimating equation between SSDE and NSSDE groups in superior and inferior quadrants

**∆: Generalized estimating equation between FL (−) and FL (+). patients in four different quadrants

**‡: Generalized estimating equation between FL (−) and FL (+). patients in superior and inferior quadrants

a: Generalized estimating equation between FL (−) and FL (+) in SSDE and NSSDE groups respectively

b: Generalized estimating equation between SSDE and NSSDE groups in FL (−) and FL (+) patients respectively

c: Generalized estimating equation between superior quadrants and inferior quadrants in SSDE and NSSDE groups respectively

d: Generalized estimating equation between superior quadrants and inferior quadrants in FL (−) and FL (+) patients respectively

e: Generalized estimating equation between superior quadrants and inferior quadrants

### OSDI values, tear production, tear film stability and corneal fluorescein staining score

The OSDI values were significantly higher in SSDE patients (56.91 ± 16.25) than in NSSDE patients (28.03 ± 13.62) (*P < .001*). The values of the Schirmer I test, tear meniscus height, FBUT and first noninvasive keratograph tear film break-up time (f-NIKBUT) in SSDE were 62, 32, 50 and 26% lower than those in NSSDE (*P < .001*; *P = .012*; *P = .001*; *P = .039;* Table 2). The average NIKBUT (av-NIKBUT) was similar between SSDE and NSSDE (*P = .193*). The corneal fluorescein staining scores were 4-fold higher in SSDE than in NSSDE patients (3.61 ± 4.46 vs 0.16 ± 0.57, *P < .001*) (Table 2). The number of patients with the absence of fluorescein staining in SSDE was almost 4 times higher than that in NSSDE (29/44 eyes vs 6/44 eyes, *P < .001*) (Table 1).

**Table 2:** Demographic Data and Clinical Characteristics of SSDE and NSSDE

	NSSDE(*n* = 44)	SSDE(n = 44)	*P*
Age	53.41 ± 11.78	58.32 ± 12.11	.057
Sex	3 M/19F	2 M/20F	.644
OSDI score	13.45 ± 6.54	27.31 ± 7.80	**<.001**
Schirmer I test(mm/5 min)	9.32 ± 7.75	3.57 ± 4.35	**<.001**
TMH(mm)	.19 ± 0.08	0.13 ± 0.08	**.012**
FBUT(s)	3.43 ± 1.74	1.70 ± 1.80	**.001**
f-NIKBUT(s)	5.60 ± 3.78	4.15 ± 2.26	**.039**
Av-NIKBUT(s)	7.98 ± 4.60	6.36 ± 4.32	.193
FL score	0.16 ± 0.57	3.61 ± 4.46	**<.001**
Meibomian gland dropout rate(%)			
Upper eyelid	34.63 ± 13.47	47.52 ± 19.49	**0.001**
Lower eyelid	49.57 ± 23.49	69.24 ± 20.69	**<0.001**

*TMH* Tear meniscus height, *FBUT* Fluorescein break-up time, *f-NIKBUT* First-noninvasive keratograph tear film break-up time, *Av-NIKBUT* Average-noninvasive keratograph tear film break-up time, *FL score* Fluorescein staining score

Generalized estimating equation was used to adjusted the correlation between right and left eyes

### The relationship between FTBUP and fluorescein staining

As shown in Table 2, the locations of FTBUP had difference between eyes with and without corneal fluorescein staining (*P = .039*). The eyes with the presence of fluorescein staining (91.5%) had a higher proportion of FTBUP occurring in the inferior quadrant than those without staining (67.9%) (*P = .011*). In SSDE with the presence of fluorescein staining, FTBUP occurred more frequently in the temporal quadrants (17 eyes, 58.6%). However, FTBUP was more likely to be seen in nasal quadrants in those with the absence of fluorescein staining (8 eyes, 60.0%) (*P = .045*). Nevertheless, the presence or absence of corneal fluorescein staining did not affect the location of FTBUP in NSSDE patients (Table 1).

### Meibomian gland dropout

**Fig. 2:**
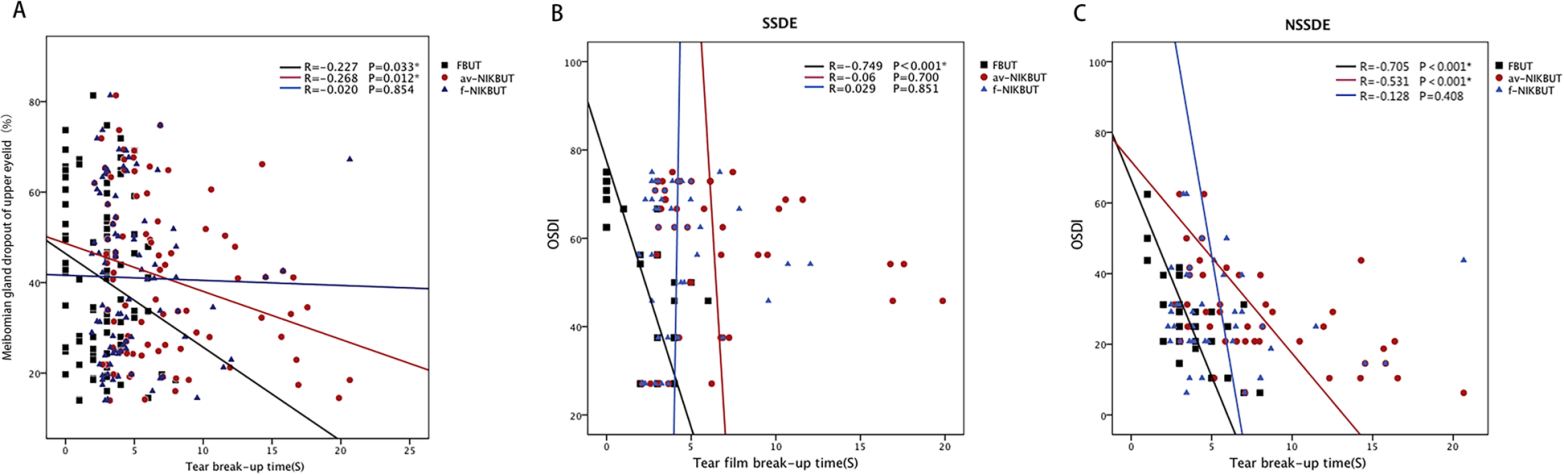
The correlation analysis on dry eye examinations. **A** Meibomian gland dropout of upper eyelid had negative correlation with fluorescein break-up time and average noninvasive keratograph tear film break-up time (*R = -.227, P = .033; R = -.268, P = .012*), but no correlation with first noninvasive keratograph tear film break-up time (*R = -.020, P = .854*). **B** In Sjögren’s Syndrome Dry Eye patients, OSDI had a negative correlation with fluorescein break-up time (*R = -.749, P<.001*) but no correlation with both first noninvasive keratograph tear film break-up time and average noninvasive keratograph tear film break-up time (*R = -.029, P = .851 and R = -.060, P = .700*). **C** In non-Sjögren’s Syndrome Dry Eye patients, OSDI had a negative correlation with fluorescein tear film break-up time and average noninvasive keratograph tear film break-up time (*R = -.705, P<.001; R = -.531, P<.001*), but no correlation with first noninvasive keratograph tear film break-up time (*R = -.128, P = .408*)

The meibomian gland dropout rate in SSDE (upper 47.52 ± 19.49% and lower 69.24 ± 20.69%) was significantly higher than that in NSSDE (upper 34.63 ± 13.47% and lower 49.57 ± 23.49%) (*P = .001and<0.001*, respectively) (Table 2). The dropout of the lower eyelid was more severe than that of the upper eyelid in both groups (SSDE: upper 47.52 ± 19.49% vs lower 69.24 ± 20.69%, *P < .001*, NSSDE: upper 34.63 ± 13.47% vs lower 49.57 ± 23.79%, *P = .001*). Meibomian gland dropout of the upper eyelid, which was mostly found at the temporal part in both SSDE (18 eyes, 40.9%) and NSSDE (27 eyes, 61.4%) (Table 3), had a negative correlation with FBUT (*R = -.227, P = .033)* and av-NIKBUT (*R = -.268, P = .012*). No correlation was found between meibomian gland dropout and f-NIKBUT (Fig. 2A).

**Table 3:** The distribution of meibomian gland dropout in SSDE and NNSDE

	Position of eyelid	NSSDE(%)	SSDE(%)	*P* value^*^
MG dropout of upper eyelid	Nasal part	6 (13.6%)	10 (22.7%)	.715
	Central part	4 (9.1%)	4 (9.1%)	
	Temporal part	27 (61.4%)	18 (40.9%)	
	Undifferentiated	7 (15.9%)	12 (27.3%)	
MG dropout of lower eyelid	Nasal part	13 (29.5%)	13 (29.5%)	.591
	Central part	0 (0%)	1 (2.3%)	
	Temporal part	7 (15.9%)	2 (4.5%)	
	Undifferentiated	24 (54.6%)	28 (63.7%)	
P value^**^		**<.001**	**<.001**	

*MG* Meibomian gland

*: Generalized estimating equation of MG dropout between SSDE and NSSDE groups in upper and lower eyelid

**: Generalized estimating equation of MG dropout between upper eyelid and lower eyelid in NSSDE and SSDE groups

### The correlations between OSDI values and dry eye examinations

The OSDI values showed a strong negative correlation with FBUT in both SSDE (*R = -.749, P < .001*) and NSSDE (*R = -.705, P < .001*). A moderate negative correlation between the OSDI values and av-NIKBUT was observed in NSSDE (*R = -.531, P < .001*). However, no significant correlation was found between the OSDI and f-NIKBUT in the two groups (Fig. 2B, C). In addition, the OSDI values had a weak positive correlation with corneal fluorescein staining scores in SSDE (*R = .306, P = .044*), which was not found in NSSDE (*R = .112, P = .471*). There was no significant correlation between the OSDI values and the Schirmer I test values and meibomian gland dropout in either SSDE or NSSDE groups.

## Discussion

In this study, we found that both SSDE and NSSDE patients had a more frequent FTBUP in the inferior quadrants, and the presence of corneal fluorescein staining affect the location of FTBUP in SSDE patients.

SSDE patients usually have more severe discomfort symptoms and ocular surface damage than NSSDE patients according to previous studies [9–14]. The current study obtained similar results: SSDE patients had higher OSDI values, less tear production, more unstable tear film, more severe corneal staining, and even a higher rate of meibomian gland dropout.

As far as FTBUP was concerned, we found that it predominantly occurred in the inferior quadrant, which was seen in 90% eyes of SSDE and 70% of NSSDE. This result was similar to previous studies focused on aqueous-deficient dry eye and meibomian gland dysfunction [19, 20]. Three possible reasons might contribute to the predominance of FTBUP in the inferior area. First, the inferior region had a thinner tear film than the superior region [29–31], which was more likely to break up earlier. Second, subbasal nerve abnormalities caused by long-term ocular surface inflammation, especially in dry eye patients, are characterized with reduced density, abnormal morphology, impaired corneal sensation and abnormal blinking [32, 33]. Reduced blinking rate and incomplete blinking contributed to overexposure of the ocular surface, especially the interpalpebral area, and increased tear evaporation [34]. Third, a higher rate of meibomian gland dropout of the lower eyelid caused reduced lipid production in the inferior region, which also contributed to the instability of the inferior tear film. Therefore, FTBUP was more likely to occur in this area in both SSDE and NSSDE groups.

The current study showed a higher proportion of eyes with the presence of fluorescein staining and higher fluorescein staining scores in SSDE, which was in agreement with previous studies [13, 35]. Moreover, in SSDE subjects, temporal breakup point was seen more often in those who presented corneal fluorescein staining in any location, while nasal breakup point was more frequent in those who did not present any corneal fluorescein staining. The presence of fluorescein staining indicated damaged barrier function of corneal epithelium due to abnormal epithelial metabolism and turnover [36–38], which lead to the infiltration of fluorescein, an unsmooth corneal surface and an unstable tear film. Nevertheless, it is out of our expectation that no significant correlation was found between the FTBUP and the location of fluorescein staining. It has been reported that tear film thinning and break-up was affected by three different directions of flows: evaporation, flow through the corneal surface and “tangential flow” along the corneal surface [29], which was determined by tear film lipid layer, the integrity of corneal epithelium, normal eyelid structure and blinking. Therefore, the location of fluorescein staining might not be the key factor to determine the location of FTBUP.

The tear film serves as the most anterior refractive surface, playing an important role in maintaining optical quality [39–41]. Many studies have shown that irregular astigmatism and wavefront aberrations caused by an unstable tear film are increased in dry eye patients [42, 43]. A short tear film break-up time increased the post-blink higher order aberrations (HOAs) and ocular forward light scattering, leading to “fluctuated vision” or “glare” [44]. Liu et al. [45] reported that irregularity of the ocular surface was positively correlated with corneal fluorescein staining. Our previous study showed that SSDE patients had worse visual quality [46]. The current study confirmed that tear film instability and ocular surface damage might be possible reasons. Nevertheless, the impact of different locations of FTBUP on visual quality needs further investigation.

Several limitations of the current study should be addressed. First, the OSDI questionnaire only evaluated ocular symptoms within the most recent week. However, most SSDE patients in our study had a long course of disease and might have decreased corneal sensation. The symptoms evaluated by the OSDI questionnaire may not be consistent with the ocular surface signs. Second, 90.9% of SSDE in the present study were not newly diagnosed, and 86.3% had already had immunosuppressant treatment such as cyclosporin A. Different treatment regimens might affect the results. The sample size was also limited. Further study with a larger number of newly diagnosed SSDE is needed to verify the findings of the current study and to investigate the impact of different locations of FTBUP on visual quality.

## Conclusion

FTBUP was more likely to occur in the inferior quadrant in both SSDE and NSSDE. The location of FTBUP in SSDE had a close relationship with the presence of corneal fluorescein staining. Hence, the maintenance of an intact and healthy corneal epithelium is crucial to a stable tear film and good visual quality. Nevertheless, the application of FTBUP to help the diagnosis of SSDE still needs confirmation by large-scale studies.

## Data Availability

The datasets used and/or analyzed during the current study are available from the corresponding author on reasonable request.

## Abbreviations

FTBUP: First tear film break-up point
SSDE: Sjögren’s Syndrome Dry Eye
NSSDE: Non-Sjögren’s Syndrome Dry Eye
FBUT: Fluorescein tear film break-up time
NIKBUT: Noninvasive keratograph tear film break-up time
f-NIKBUT: first-Noninvasive keratograph tear film break-up time
av-NIKBUT: Average-Noninvasive keratograph tear film break-up time
